# The mutation in splicing factor genes correlates with unfavorable prognosis, genomic instability, anti-tumor immunosuppression and increased immunotherapy response in pan-cancer

**DOI:** 10.3389/fcell.2022.1045130

**Published:** 2023-01-06

**Authors:** Jiangti Luo, Canping Chen, Zhixian Liu, Xiaosheng Wang

**Affiliations:** ^1^ Biomedical Informatics Research Lab, School of Basic Medicine and Clinical Pharmacy, China Pharmaceutical University, Nanjing, China; ^2^ Cancer Genomics Research Center, School of Basic Medicine and Clinical Pharmacy, China Pharmaceutical University, Nanjing, China; ^3^ Big Data Research Institute, China Pharmaceutical University, Nanjing, China; ^4^ Jiangsu Cancer Hospital, Jiangsu Institute of Cancer Research, The Affiliated Cancer Hospital of Nanjing Medical University, Nanjing, Jiangsu, China

**Keywords:** somatic mutations, splicing factor genes, pan-cancer, cancer prognosis, anti-tumor immune responses, cancer immunotherapy, genomic instability

## Abstract

Splicing abnormality resulting from somatic mutations in key splicing factor genes (SFG) has been detected in various cancers. Hence, an in-depth study of splicing factor genes mutations’ impact on pan-cancer is meaningful. This study investigated associations of splicing factor genes mutations with clinical features, tumor progression phenotypes, genomic integrity, anti-tumor immune responses, and immunotherapy response in 12 common cancer types from the TCGA database. Compared to SFG-wildtype cancers, SFG-mutated cancers displayed worse survival prognosis, higher tumor mutation burden and aneuploidy levels, higher expression of immunosuppressive signatures, and higher levels of tumor stemness, proliferation potential, and intratumor heterogeneity (ITH). However, splicing factor genes-mutated cancers showed higher response rates to immune checkpoint inhibitors than splicing factor genes-wildtype cancers in six cancer cohorts. Single-cell data analysis confirmed that splicing factor genes mutations were associated with increased tumor stemness, proliferation capacity, PD-L1 expression, intratumor heterogeneity, and aneuploidy levels. Our data suggest that the mutation in key splicing factor genes correlates with unfavorable clinical outcomes and disease progression, genomic instability, anti-tumor immunosuppression, and increased immunotherapy response in pan-cancer. Thus, the splicing factor genes mutation is an adverse prognostic factor and a positive marker for immunotherapy response in cancer.

## Introduction

RNA splicing is a dynamic process in which pre-mRNA exon-intron borders are identified, and the intervening intronic sequences are removed, leaving coding exons ligated to form mature mRNA (Witten and Ule, 2011). Alternative pre-mRNA splicing is a major cause of transcript diversity in mammalian cells, which is orchestrated by the spliceosome, a megadalton complex to generate tissue- and species-specific differentiation patterns (Papasaikas and Valcarcel, 2016). The spliceosome comprises five small nuclear ribonucleoproteins (snRNPs) and numerous auxiliary proteins (Matera and Wang, 2014). There are two classes of introns: U2-type and U12-type. The U2-type accounts for > 99.5% of introns, while the U12-type is responsible for < .5% of introns and resides in around 700–800 genes. The introns are spliced by U2-type- and U12-type-dependent spliceosomes, also known as “major” and “minor” spliceosomes, respectively (Akinyi and Frilander, 2021). Although the functional roles of most isoforms generated by alternative splicing remain unclear, specific isoforms are selected in cancer to promote neoplastic transformation, cancer progression, and drug resistance (David and Manley, 2010; Zhang and Manley, 2013). In some cases, recurrent somatic point mutations near splice sites may contribute to specific splicing changes that promote cancer development by inducing mis-splicing of tumor suppressor genes (Supek et al., 2014; Jung et al., 2015).

Hotspot mutations in splicing factor genes (SFG) have been recently reported to have a high frequency in hematological malignancies and solid tumors (Patnaik et al., 2013; Landau et al., 2015). It suggests the importance of RNA splicing in cancer. Recurrent somatic mutations of the SFG, such as *SF3B1*, *SRSF2*, *U2AF1*, and *ZRSR2*, have been uncovered in myelodysplastic syndrome (MDS) (Yoshida et al., 2011), chronic lymphocytic leukemia (Mansouri et al., 2013), acute myeloid leukemia (Cancer Genome Atlas Research et al., 2013), breast cancer (McCullough, 2013), lung adenocarcinoma (Imielinski et al., 2012), and uveal melanoma (Harbour et al., 2013). Given the crucial roles of specific alternatively spliced isoforms in cancer development and the increased sensitivity of cancer cells to global perturbation of splicing efficiency *versus* normal cells, the pharmacological intervention of splicing may represent an important therapeutic strategy (Hubert et al., 2013; Hsu et al., 2015). Because increased tumor mutation burden (TMB) may generate neoepitopes to render a specific subpopulation of cancer patients sensitive to immunotherapies (Chan et al., 2015; Rizvi et al., 2015), abnormal mRNAs generated by mutations in spliceosomal genes can result in neoepitope production in cancers. Seiler et al. (Seiler et al., 2018) have reported that 119 SFGs have putative driver mutations in 33 TCGA cancer types. They found that the most common mutations were mutually exclusive and were associated with lineage-independent altered splicing. Furthermore, tumors with these mutations were deregulated in cell-autonomous pathways and immune infiltration (Seiler et al., 2018).

Nevertheless, an in-depth study of SFG mutations’ impact on pan-cancer remains insufficient. With the recent development of next-generation sequencing and single-cell sequencing technologies, many cancer-specific multi-omics data have been generated, which provides a unique opportunity to interrogate splicing deregulation due to somatic mutations across human cancers. Here we performed a systematic analysis of the association between SFG mutations and alterations of molecular and clinical features in 12 TCGA cancer types, including those most common human cancer types, such as breast, lung, colon, stomach, and prostate cancers. We also validated our findings in tumors by analyzing single-cell transcriptomes.

## Methods

### Datasets

We downloaded transcriptome (RNA-Seq, RSEM normalized), somatic mutations, somatic copy number alterations (SCNAs), protein expression profiling, and clinical data for 12 TCGA cancer types from the genomic data commons (GDC) data portal (https://portal.gdc.cancer.gov/). The 12 cancer types included adrenocortical carcinoma (ACC), bladder urothelial carcinoma (BLCA), breast invasive carcinoma (BRCA), colon adenocarcinoma (COAD), head and neck squamous cell carcinoma (HNSC), kidney renal clear cell carcinoma (KIRC), lung adenocarcinoma (LUAD), lung squamous cell carcinoma (LUSC), prostate adenocarcinoma (PRAD), skin cutaneous melanoma (SKCM), stomach adenocarcinoma (STAD), and uterine corpus endometrial carcinoma (UCEC). We obtained 119 key splicing factor genes from the publication by Seiler et al. (Seiler et al., 2018). Moreover, we obtained the datasets for six cancer cohorts treated with ICIs, which involved somatic mutation profiles and immunotherapy response data in cancer patients. These datasets included Hugo (melanoma) (Hugo et al., 2016), Riaz (melanoma) (Riaz et al., 2017), Hellmann (non-small cell lung cancer) (Hellmann et al., 2018), Liu (metastatic melanoma) (Liu et al., 2019), Rizvi (non-small cell lung cancer) (Rizvi et al., 2015), and Lauss (melanoma) (Lauss et al., 2017) cohorts. We defined a tumor as SFG-mutated if at least one of the 119 key splicing factor genes mutated in the tumor; otherwise, the tumor was defined as SFG-wildtype. In addition, we obtained six single-cell RNA sequencing (scRNA-seq) transcriptomic datasets for six cancer cohorts from the NCBI gene expression omnibus (GEO) (https://www.ncbi.nlm.nih.gov/geo/). These cancer cohorts included BRCA (Qian et al., 2020), COAD (Qian et al., 2020), SKCM (Jerby-Arnon et al., 2018), PRAD (Chen et al., 2021), LUAD (Maynard et al., 2020), and HNSC (Puram et al., 2017). Prior to subsequent analyses, we normalized all gene expression values by log_2_ (RSEM or TPM + 1). A summary of these datasets is shown in Supplementary Table S1.

### Gene-set enrichment analysis

We used the single-sample gene-set enrichment analysis (ssGSEA) (Hänzelmann and Guinney, 2013) to score the enrichment of pathways, immune signatures, proliferation, and stemness signatures based on the expression profiles of their pathway or marker genes. The pathway genes were obtained from KEGG (Kanehisa et al., 2017), and the marker genes of immune, proliferation and stemness signatures were obtained from several publications, including CD8^+^ T cells (Li et al., 2020a), proliferation (Li et al., 2020a), and tumor stemness (Miranda et al., 2019).

### Calculation of the ratio of two immune signatures

The ratio of two immune signatures in a tumor is the log2-transformed value of the geometric mean expression level of all marker genes in an immune signature over that in another immune signature.

### Evaluation of TMB, homologous recombination deficiency (HRD) and SCNAs

We defined a tumor’s TMB as the total number of somatic mutations detected in the tumor. From the publication by Knijnenburg et al. (2018), we obtained HRD scores (aneuploidy levels) of TCGA cancers. Knijnenburg et al. defined HRD scores in 9,125 TCGA cancer samples based on HRD loss of heterozygosity, large-scale state transitions, and the number of telomeric allelic imbalances. We utilized GISTIC2 (Mermel et al., 2011) to calculate G-scores of SFG-mutated and SFG-wildtype tumors with the input of “SNP6” files, representing the amplitude and frequency of SCNAs across a group of samples.

### Logistic regression analysis

We predicted the ratios of two immune signatures (high (>median) *versus* low (<median)) by the logistic regression model with three predictors (SFG mutation, HRD score, and TMB). The SFG mutation was a discrete variable, and the HRD score and TMB were continuous variables. In the logistic regression analysis, we first normalized all values by z-score and then fitted the binary model with the “glm ()” R function. In the function, the parameter “family” was specified as “binomial” and other parameters as default.

### Survival analysis

We utilized Kaplan–Meier curves to display the survival time differences between different groups of cancer patients. A total of three survival endpoints were analyzed, including overall survival (OS), progression-free interval (PFI), and disease-specific survival (DSS). The log-rank test was used to assess the significance of survival time differences.

### Single-cell data pre-process and visualization

We utilized the R package “Seurat” (v3.2.1) (Butler et al., 2018) to pre-process the scRNA-seq data before subsequent analyses. We first filtered out the genes expressed in less than three tumor cells and deleted the cells expressing less than 200 genes. Next, we normalized the gene expression matrix using the function “NormalizeData” with the default parameters. Furthermore, we identified the genes whose expression had high variations across single cells by the function “FindVariableFeatures” with the default “vst” method in “Seurat.” Based on these genes, we performed the principal component analysis of the gene expression matrix. With the first ten principal components, we built a shared nearest neighbor graph to cluster cells using the “FindClusters” function. Finally, we employed the uniform manifold approximation and projection (UMAP) (Becht et al., 2018) method (the “RunUMAP” R function) to visualize cells in low dimensions.

### Inference of SFG mutations in single cells

We identified subpopulations of cancer single cells with a certain phenotype, such as SFG mutations, using the Scissor algorithm (Sun et al., 2022). Scissor defines phenotype-associated subpopulations of single cells by integrating bulk transcriptomes with phenotypic features and single-cell transcriptomes based on the similarity between single cell and bulk tumor expression profiles.

### Inference of DNA copy number variations (CNVs) in single cells

We utilized inferCNV (Patel et al., 2014) to infer CNVs in cancer cells relative to normal cells. We converted all CNV values inferred by inferCNV to 0, 1, or 2, where “0” indicates neutral, “1” loss or gain of one copy, and “2” loss or gain of two copies. The re-standardized CNV score of each cell was the sum of the CNV value for each gene.

### Clustering single cells

We used the single-cell consensus clustering (SC3) algorithm (Kiselev et al., 2017) to perform unsupervised clustering of single cells.

### Statistical analysis

In comparisons of two classes of samples, we used the Student’s *t*-test if they followed normal distributions; otherwise, we used the Mann–Whitney *U* test. We employed the Fisher’s exact test to examine the correlation between two categorical variables and the Z-test to compare the population proportions between two groups. We performed all statistical analyses in the R programming environment (version 3.6.1).

## Results

### Mutations of SFG are associated with unfavorable clinical outcomes in cancer

**FIGURE 1:**
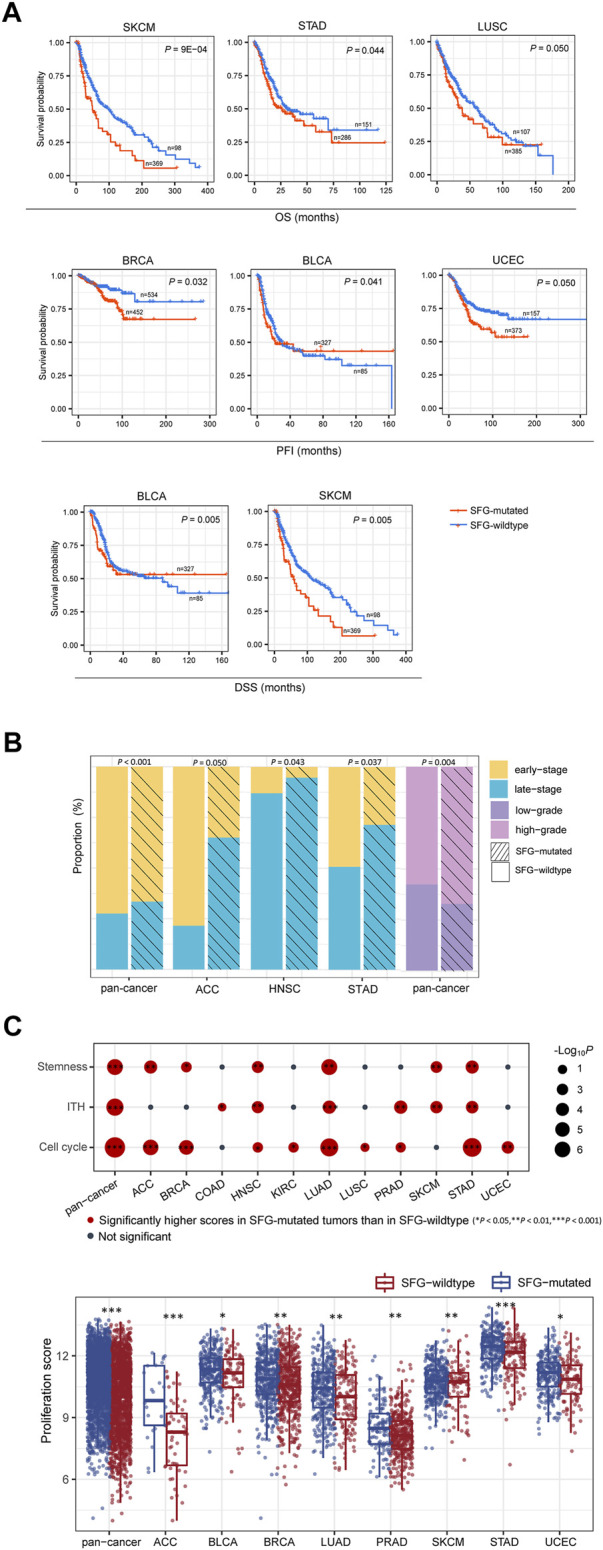
SFG mutations correlate with unfavorable clinical outcomes in cancer. **(A)** Kaplan-Meier curves displaying that SFG-mutated tumors have worse survival prognosis than SFG-wildtype tumors in multiple cancer types. The log-rank test *p* values are shown. **(B)** Advanced (late-stage or high-grade) tumors include a significantly higher proportion of SFG-mutated tumors than non-advanced (early-stage or low-grade) tumors in pan-cancer and in multiple cancer types. The Fisher’s exact test *p* values are shown. **(C)** The tumor progression phenotypes (stemness, proliferation, intratumor heterogeneity (ITH), and cell cycle activity) have significantly higher scores in SFG-mutated than in SFG-wildtype tumors in pan-cancer and in multiple cancer types. The one-tailed Mann-Whitney *U* test *p* values are shown.

We found that in six cancer types (BLCA, BRCA, LUSC, SKCM, STAD, and UCEC), mutations of SFG were correlated with worse OS, DSS, and/or PFI (log-rank test, *p* ≤ .05) (Figure 1A). Furthermore, we found that late-stage (stage III-IV) tumors harbored a significantly higher proportion of SFG-mutated tumors compared with early-stage (stage I-II) tumors in pan-cancer (Fisher’s exact test, *p* < .001) and in three individual cancer types (ACC, HNSC, and STAD) (*p* < .05) (Figure 1B). Tumor grade represents the growth speed and spread extent of a tumor on the basis of the abnormality degree of tumor cells relative to normal cells. We found that SFG-mutated tumors constituted a higher proportion in high-grade (G3-4) than in low-grade (G1-2) tumors in pan-cancer (*p* = .004) (Figure 1B).

We further compared several phenotypes indicating tumor progression or unfavorable prognosis, including tumor stemness, proliferation, and intratumor heterogeneity (ITH). Notably, all these phenotypes showed significantly higher scores in SFG-mutated than in SFG-wildtype tumors in pan-cancer (one-tailed Mann-Whitney *U* test, *p* < .001) (Figure 1C). Moreover, in 6, 8, and 6 individual cancer types, SFG-mutated tumors displayed significantly higher scores of stemness, proliferation, and ITH than SFG-wildtype tumors, respectively (Figure 1C). In addition, SFG-mutated tumors showed significantly higher enrichment scores of the cell cycle pathway than SFG-wildtype tumors in pan-cancer and in nine individual cancer types (Figure 1C).

Taken together, these results support that SFG mutations are associated with unfavorable clinical outcomes in various cancers.

### Mutations of SFG are associated with genomic instability in cancer

**FIGURE 2:**
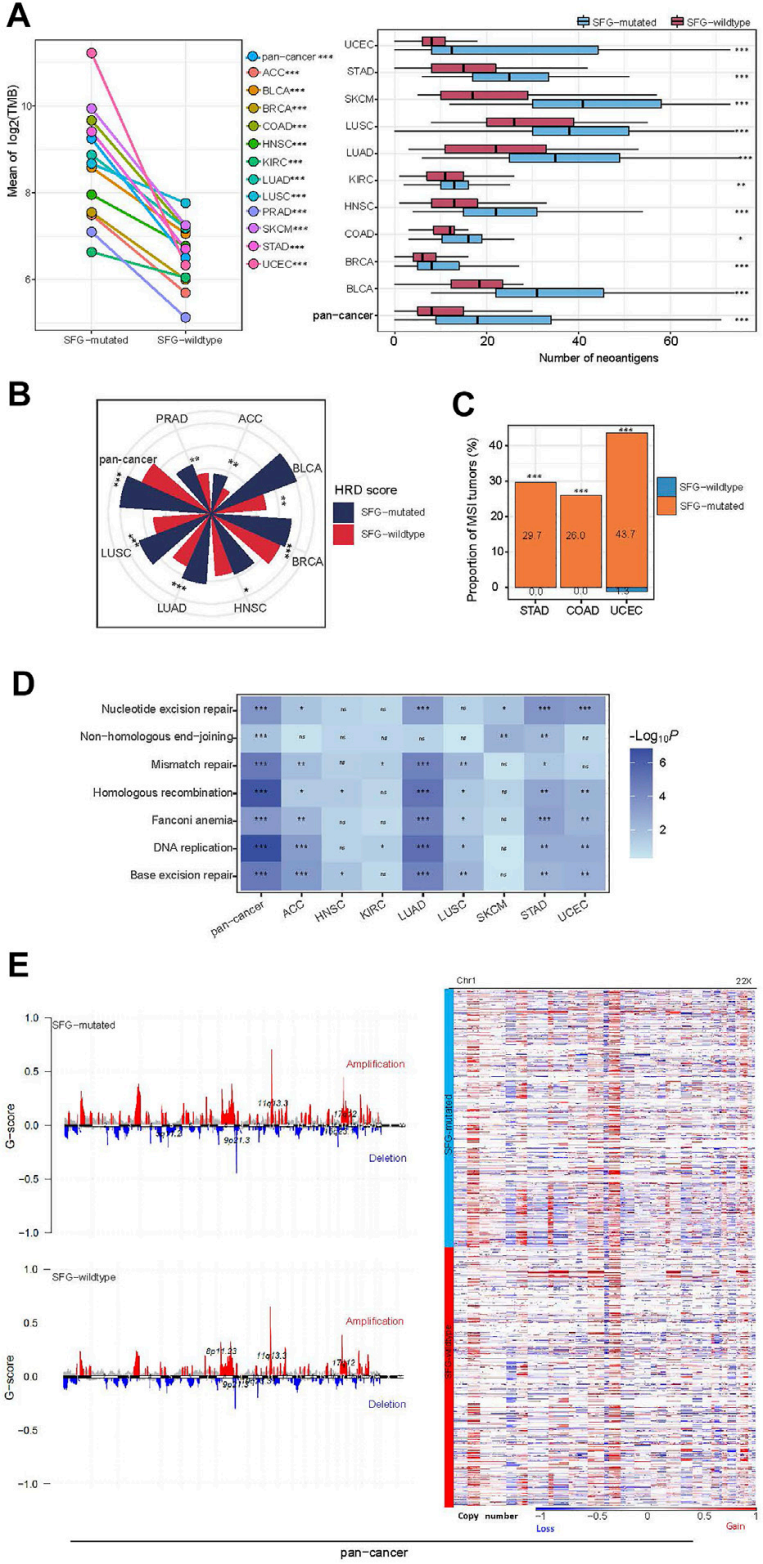
SFG mutations correlate with genomic instability in cancer. **(A)** SFG-mutated tumors have significantly higher tumor mutation burden (TMB) and neoantigens than SFG-wildtype tumors in pan-cancer and in most individual cancer types. **(B)** SFG-mutated tumors have significantly higher homologous recombination deficiency (HRD) scores than SFG-wildtype tumors in pan-cancer and in seven individual cancer types. **(C)** SFG-mutated tumors harbor a significantly higher proportion of MSI tumors than SFG-wildtype tumors in the cancer types with a high prevalence of MSI tumors. **(D)** SFG-mutated tumors have higher enrichment of DNA repair pathways than SFG-wildtype tumors. **(E)** SFG-mutated tumors have higher levels of copy number alteration (G-scores) than SFG-wildtype tumors in pan-cancer, and heatmap shows that SFG-mutated tumors have higher amplitudes of copy number amplification and deletion across chromosomes than SFG-wildtype tumors. The one-tailed Mann-Whitney *U* test *p* values are shown in **(A,B,D)**, and the Fisher’s exact test *p* values are shown in **(C)**. *p < .05, **p < .01, ***p < .001, ^ns^ not significant.

Genomic instability often results in increased TMB and CNAs (Li et al., 2020b). Notably, SFG-mutated tumors had significantly higher TMB than SFG-wildtype tumors in pan-cancer and all the 12 cancer types (one-tailed Mann-Whitney *U* test, *p* < .001) (Figure 2A). As expected, SFG-mutated tumors harbored far more neoantigens (Rooney et al., 2015) than SFG-wildtype tumors in pan-cancer and in 10 individual cancer types (*p* < .05) (Figure 2A). HRD may lead to large-scale genomic instability, namely aneuploidy (Matera and Wang, 2014). We found that SFG-mutated tumors had significantly higher HRD scores (i.e., aneuploidy levels) than SFG-wildtype tumors in pan-cancer and in eight individual cancer types (one-tailed Mann-Whitney *U* test, *p* < .05) (Figure 2B). DNA mismatch repair deficiency (dMMR) or microsatellite instability (MSI) is a prevalent pattern of genomic instability in certain cancer types, such as endometrial, colorectal, and gastric cancers. We found that SFG-mutated tumors harbored a significantly higher proportion of MSI tumors than SFG-wildtype tumors in UCEC, COAD, and STAD (Fisher’s exact test, *p* < .001) (Figure 2C). In addition, we found many DNA repair pathways showing higher enrichment in SFG-mutated than in SFG-wildtype tumors in at least a half of the 12 cancer types. These pathways included DNA replication, base excision repair, nucleotide excision repair, non-homologous end-joining, Fanconi anemia, homologous recombination, and mismatch repair (Figure 2D). Furthermore, the amplitude and frequency of SCNAs, including copy number amplifications and deletions, were higher in SFG-mutated than in SFG-wildtype tumors in pan-cancer across the chromosome (Figure 2E). These results collectively suggest a significant association between SFG mutations and increased genomic instability in diverse cancers.

Interestingly, we found smoking to have a significant positive correlation with SFG mutations, as evidenced by the fact that smokers had a significantly higher proportion of SFG mutations than non-smokers in pan-cancer (75.09% *versus* 55.52%; *Z*-test, *p* = 5.8 × 10^–13^). The reason explaining this result could be that smoking can incite mutations of tumor cells to enhance TMB (Yoshida et al., 2020).

### Mutations of SFG are associated with reduced anti-tumor immune responses and increased immunotherapy response

**FIGURE 3:**
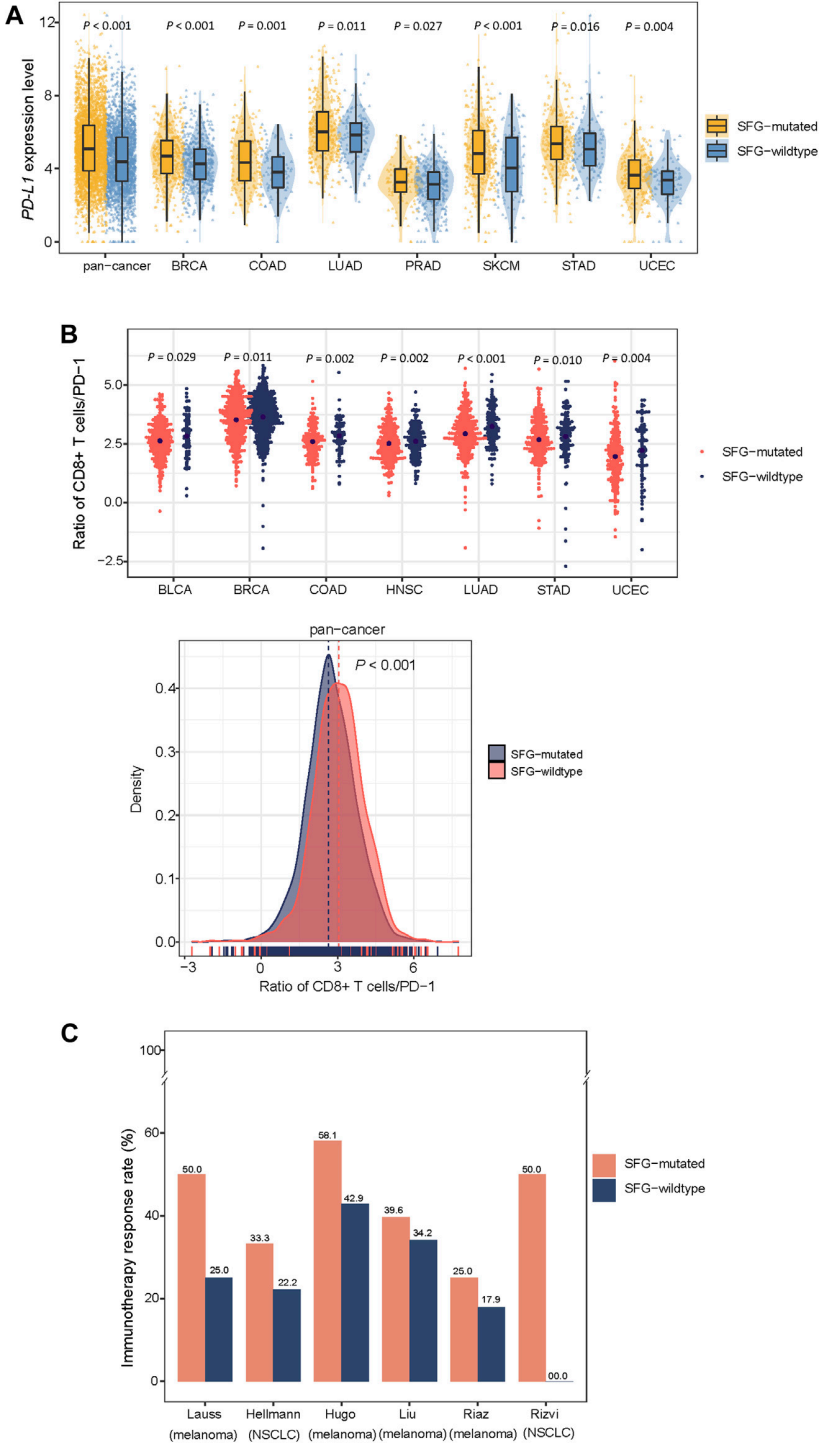
SFG mutations correlate with reduced anti-tumor immune responses and increased immunotherapy response in cancer. SFG-mutated tumors have higher expression levels of *PD-L1* **(A)** and the ratios of immune-stimulatory to immune-inhibitory signatures (CD8^+^ T cells/PD-1) **(B)** than SFG-wildtype tumors in pan-cancer and in seven cancer types. The Student’s *t* test *p* values are shown in **(A,B)**. **(C)** SFG-mutated tumors have higher response rates to immune checkpoint inhibitors (ICIs) than SFG-wildtype tumors in six cancer cohorts.

We found that SFG-mutated tumors had significantly higher expression levels of *PD-L1* in pan-cancer and in seven individual cancer types (Student’s *t*-test, *p* < .05; FC > 1.5) (Figure 3A). Furthermore, in pan-cancer and in seven individual cancer types, the ratios of immune-stimulatory to immune-inhibitory signatures (CD8^+^ T cells/PD-1) were significantly lower in SFG-mutated than in SFG-wildtype tumors (*p* < .05) (Figure 3B). Because both TMB and tumor aneuploidy have significant associations with anti-tumor immune responses (Davoli et al., 2017) and meanwhile the SFG mutation has significant associations with TMB and tumor aneuploidy, the significant correlation between the SFG mutation and anti-tumor immune responses could be mediated by TMB and tumor aneuploidy. To clarify this speculation, we performed logistic regression to predict high *versus* low ratios of CD8^+^ T cells/PD-1 in pan-cancer using three predictors: SFG mutation, HRD score, and TMB. This analysis demonstrated that the SFG mutation was a significant, negative predictor of ratios of CD8^+^ T cells/PD-1 (*p* = 4.2 × 10^–8^; *β* = -.33). Taken together, these results suggest a significant association between SFG mutations and reduced anti-tumor immune responses in diverse cancers.

Because both PD-L1 expression (Patel and Kurzrock, 2015) and TMB (Goodman et al., 2017) are positive predictors of the response to immune checkpoint inhibitors (ICIs) and SFG mutations have heightened PD-L1 expression and TMB in cancer, we anticipated that SFG-mutated tumors would respond better to ICIs than SFG-wildtype tumors. As expected, in six cancer cohorts receiving ICI treatments, SFG-mutated tumors displayed higher response rates to ICIs than SFG-wildtype tumors (Figure 3C). These cohorts included Hugo (melanoma) (Hugo et al., 2016), Riaz (melanoma) (Riaz et al., 2017), Hellmann (non-small cell lung cancer) (Hellmann et al., 2018), Liu (metastatic melanoma) (Liu et al., 2019), Rizvi (non-small cell lung cancer) (Rizvi et al., 2015), Lauss (melanoma) (Lauss et al., 2017) cohorts, in which the response rates to ICIs in SFG-mutated *versus* SFG-wildtype tumors were 58.1% *versus* 42.9%, 25.0% *versus* 17.9%, 33.3% *versus* 22.2%, 39.6% *versus* 34.2%, 50% *versus* 0%, 50% *versus* 25%, respectively.

### Different cancer subtypes have significantly different mutation rates of SFG

**FIGURE 4:**
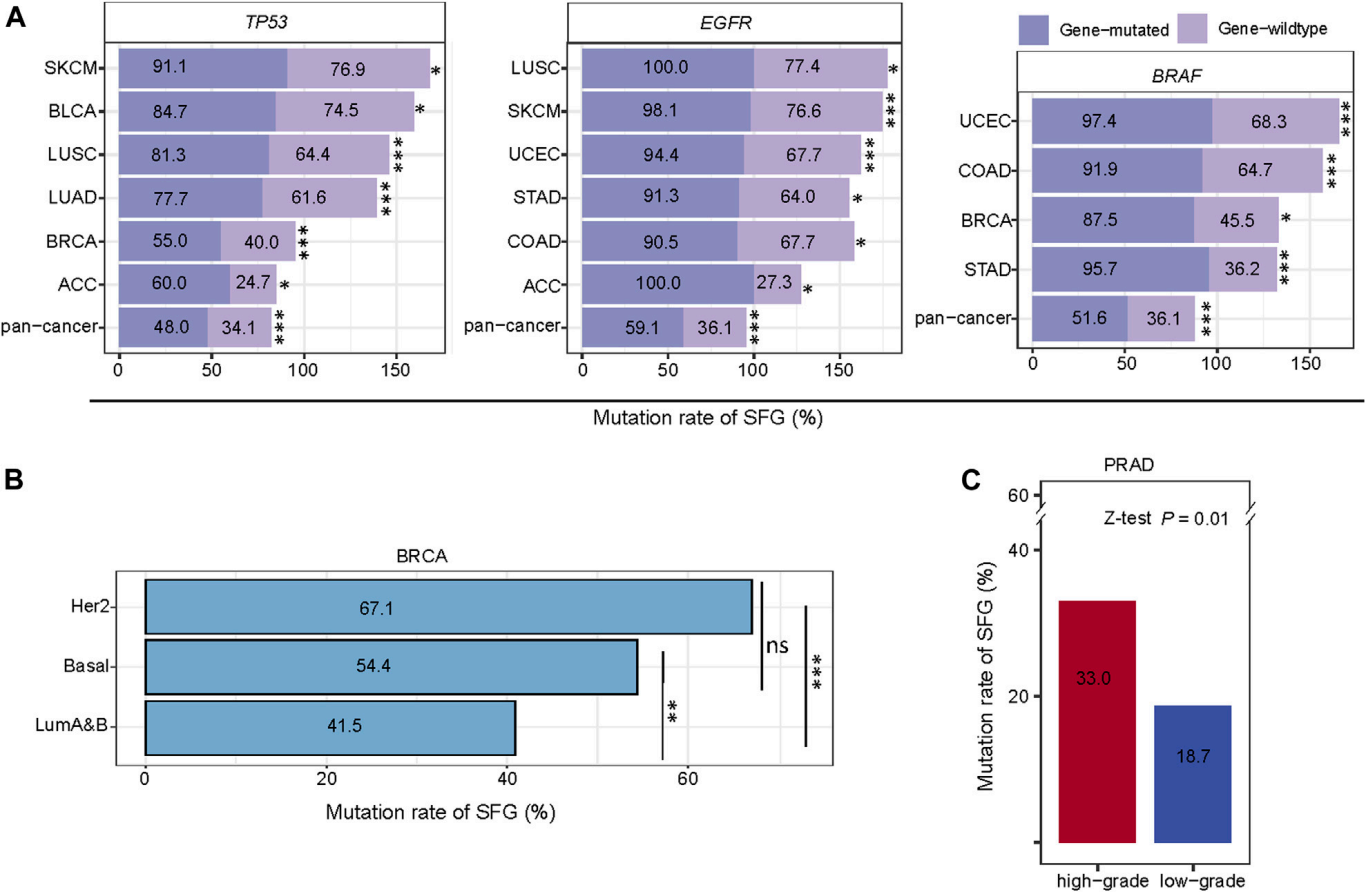
Comparisons of mutation rates of SFG between different cancer subtypes. **(A)** The tumors with mutations in *TP53*, *EGFR*, or *BRAF* have significantly higher mutation rates of SFG than the tumors without such mutations in pan-cancer and in multiple cancer types. **(B)** Comparisons of mutation rates of SFG among the breast cancer subtypes. **(C)** High-grade tumors (Gleason score > 7) have a significantly higher mutation rate of SFG than low-grade tumors (Gleason score < 7). *p < .05, **p < .01, ***p < .001, ^ns^ not significant. The Z-test *p* values are shown in **(A,B,C)**.

The mutations in certain tumor suppressor genes or oncogenes occur frequently in cancer, and their mutations are associated with cancer onset and development, such as *TP53*, *EGFR*, *KRAS*, and *BRAF* mutations. Notably, these genes-mutated tumors exhibited significantly higher mutation rates of SFG than these genes-wildtype tumors in pan-cancer and in multiple individual cancer types (Z-test, *p* < .05) (Figure 4A). For example, in pan-cancer and in six individual cancer types (ACC, BLCA, BRCA, LUAD, LUSC, and SKCM), the *TP53*-mutated subtype had higher mutation rates of SFG than the *TP53*-wildtype subtype. The *EGFR*-mutated subtype displayed higher mutation rates of SFG than the *EGFR*-wildtype subtype in pan-cancer and in six individual cancer types (ACC, COAD, LUSC, SKCM, STAD, and UCEC). SFG showed significantly higher mutation rates in *BRAF*-mutated than in *BRAF*-wildtype tumors in pan-cancer and in four individual cancer types (BRCA, COAD, STAD, and UCEC).

We further compared mutation rates of SFG among the breast cancer subtypes: luminal A&B, HER2-enriched, and basal-like, which were determined by the PAM50 assay (Parker et al., 2009). Notably, both HER2-enriched and basal-like displayed significantly higher mutation rates of SFG than luminal A&B (*p* < .01), while the SFG mutation rates showed no significant difference between HER2-enriched and basal-like (*p* = .07) (Figure 4B). Overall, these results indicate that the SFG mutation is an adverse prognostic factor in breast cancer since luminal A&B often has a better prognosis than HER2-enriched and basal-like breast cancers (Howlader et al., 2018). In PRAD, the Gleason score, ranging from 6 to 10, is the most common system to grade prostate cancer. A higher Gleason score indicates a higher grade of malignancy. We found that high-grade tumors (Gleason score > 7) have a significantly higher mutation rate of SFG than low-grade tumors (Gleason score ≤ 7) in PRAD (33.0% *versus* 18.7%; Z-test, *p* = .01) (Figure 4C).

Taken together, these results suggest that SFG was more frequently mutated in aggressive than in unaggressive cancer subtypes.

### Proteins upregulated in SFG-mutated cancers are mainly associated with cell cycle regulation and genomic instability

**FIGURE 5:**
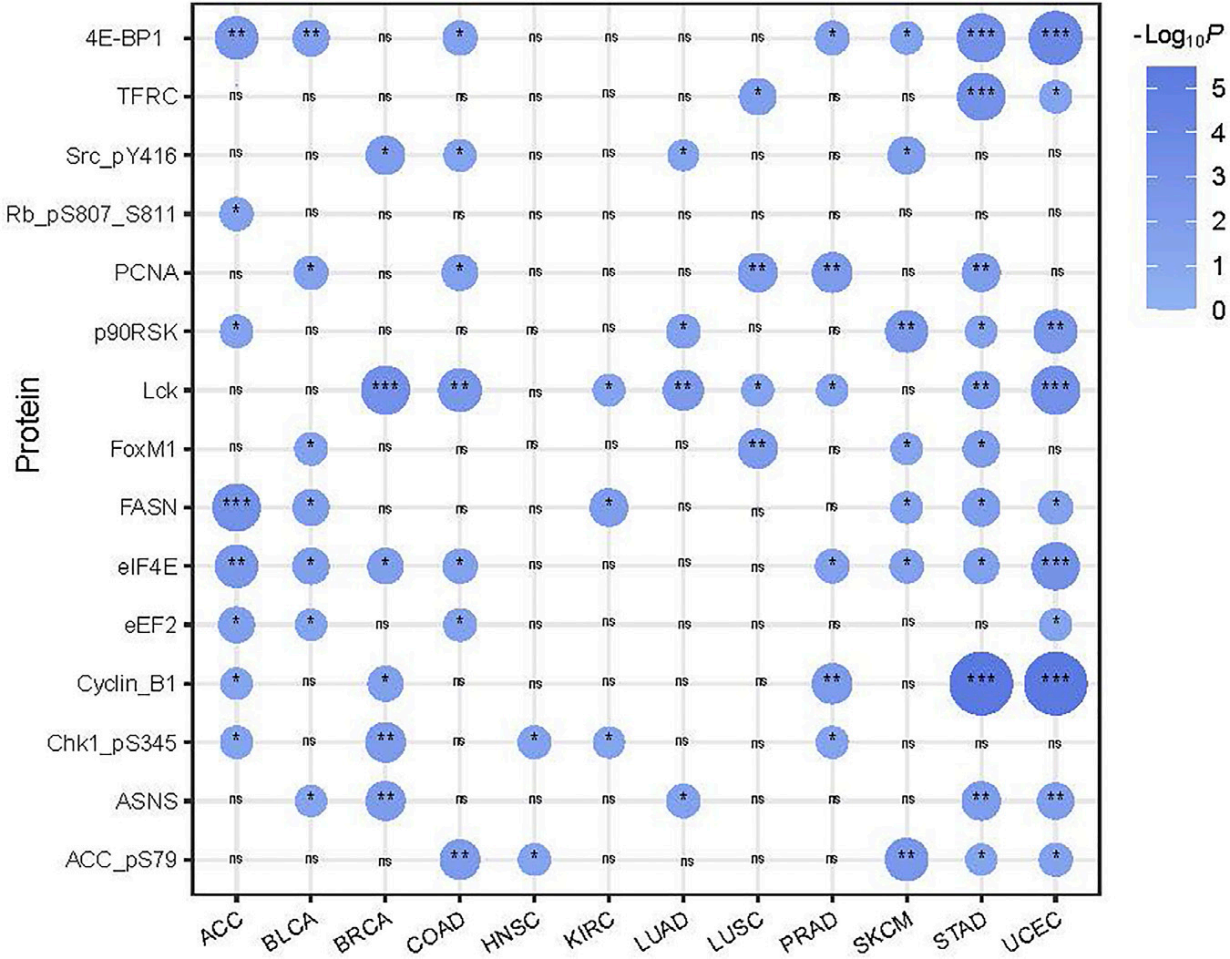
15 proteins showing significantly higher expression levels in SFG-mutated than in SFG-wildtype tumors in at least five cancer types. The two-tailed Student’s *t* test *p* values are shown.

We compared protein expression profiles between SFG-mutated and SFG-wildtype tumors in each of the 12 cancer types. We found 15 proteins having significantly higher expression levels in SFG-mutated than in SFG-wildtype tumors in at least five cancer types (two-tailed Student’s *t*-test, FDR < .05) (Figure 5). These proteins included Lck, PCNA, ASNS, 4E-BP1, FASN, ACC_pS79, Cyclin_B1, Chk1_pS345, eEF2, eIF4E, FoxM1, Rb_pS807_S811, p90RSK, Src_pY416, and TFRC. Notably, many of these proteins are cell cycle regulators, such as Cyclin_B1, Chk1_pS345, FoxM1, Rb_pS807_S811, and Src_pY416. In addition, some proteins are involved in DNA damage repair, such as PCNA. These results are concordant with the previous analysis showing that SFG mutations are associated with cell cycle activation and genomic instability.

### Exploration of SFG mutations in cancer single cells

**FIGURE 6:**
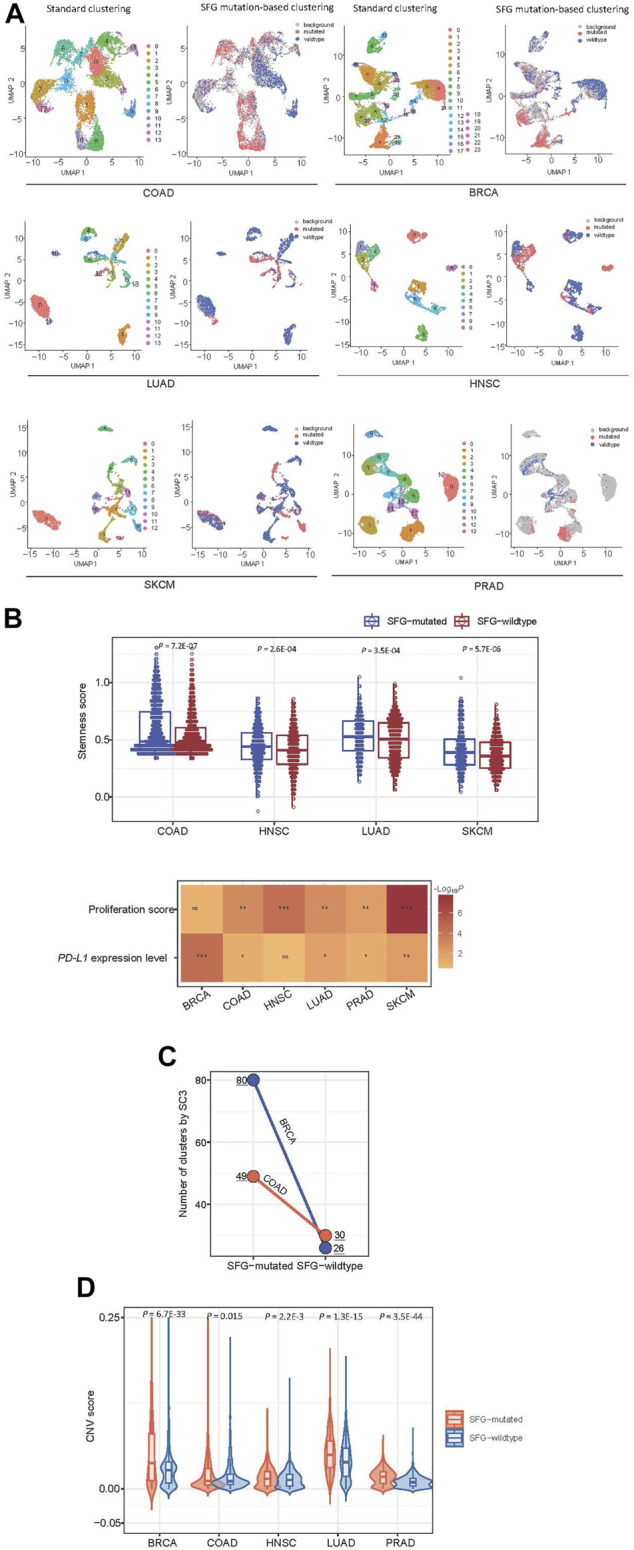
Exploration of SFG mutations in cancer single cells. **(A)** Identification of subpopulations of cancer single cells with phenotypes of SFG mutations and SFG wildtype in six single-cell transcriptomes by the Scissor algorithm (Sun et al., 2022). **(B)** The SFG-mutated cancer cell subpopulation has significantly higher scores of stemness and proliferation and higher *PD-L1* expression levels than the SFG-wildtype cancer cell subpopulation. **(C)** The single-cell consensus clustering by SC3 (Kiselev et al., 2017) identifying more clusters in SFG-mutated cell subpopulations than in SFG-wildtype cell subpopulations in two cancer types. **(D)** The SFG-mutated cell subpopulation has significantly greater CNV values than the SFG-wildtype cell subpopulation in five cancer types. The CNV values were inferred by the inferCNV algorithm (Patel et al., 2014).

Using the Scissor algorithm (Sun et al., 2022), we identified subpopulations of cancer single cells with phenotypes of SFG mutations and SFG wildtype in six single-cell transcriptomes from BRCA (Qian et al., 2020), COAD (Qian et al., 2020), SKCM (Jerby-Arnon et al., 2018), PRAD (Chen et al., 2021), LUAD (Maynard et al., 2020), and HNSC (Puram et al., 2017), respectively (Figure 6A). In 4 and five cancer types, the SFG-mutated cell subpopulation showed significantly higher scores of stemness and proliferation than the SFG-wildtype cell subpopulation, respectively (*p* < .01) (Figure 6B). In five cancer types, the SFG-mutated cell subpopulation tended to have significantly higher *PD-L1* expression levels compared to than the SFG-wildtype cell subpopulation (*p* < .05) (Figure 6B). Overall, these results are consistent with those by analyzing tumor tissues. Furthermore, we used the SC3 algorithm (Kiselev et al., 2017) to cluster cancer cells in SFG-mutated and SFG-wildtype cell subpopulations, respectively. Among two cancer types (COAD and BRCA), SC3 uncovered significantly different numbers of cell clusters between SFG-mutated and SFG-wildtype cell subpopulations (Figure 6C). In COAD, the SFG-mutated cell subpopulation involved 49 clusters *versus* 30 clusters in the SFG-wildtype cell subpopulation. In BRCA, the SFG-mutated cell subpopulation included 80 clusters *versus* 26 clusters in the SFG-wildtype cell subpopulation. It suggests that SFG-mutated cell subpopulations are more heterogeneous than SFG-wildtype cell subpopulations, supporting the higher ITH of SFG-mutated *versus* SFG-wildtype tumors. We employed the inferCNV algorithm (Patel et al., 2014) to infer CNVs in cancer cells with reference to normal cells. Notably, in five cancer types, the SFG-mutated cell subpopulation had significantly greater CNV values than the SFG-wildtype cell subpopulation (*p* < .02) (Figure 6D). These results confirmed that SFG mutations had a significant association with genomic instability at the single-cell level.

## Discussion

Splicing abnormality caused by somatic mutations in SFG has been detected in a wide variety of human cancers. In this study, we comprehensively investigated associations of SFG mutations with clinical features, genomic integrity, anti-tumor immune responses, and immunotherapy response in 12 common cancer types and their pan-cancer. Our analysis showed that SFG mutations were associated with worse clinical outcomes, higher genomic instability, and anti-tumor immunosuppression but increased immunotherapy response in diverse cancer types. In addition, SFG mutations were associated with tumor progression or unfavorable phenotypes, such as high tumor stemness, proliferation potential, and ITH. It is interesting to observe that SFG mutations are correlated with a better response to ICIs in multiple cancers, although SFG mutations have a significant association with reduced anti-tumor immune responses. Increased TMB (and thus neoantigens) and PD-L1 expression in SFG-mutated cancers could explain why this subtype responds better to ICIs than SFG-wildtype cancers since both TMB and PD-L1 expression are positive predictors of immunotherapy response (Patel and Kurzrock, 2015; Samstein et al., 2019). On the other hand, high levels of genomic instability, i.e., SCNAs or aneuploidy, may lead to reduced anti-tumor immune responses in cancers with SFG mutations since tumor aneuploidy may promote anti-tumor immune evasion (Davoli et al., 2017). Taken together, our results indicate that the relationship between anti-tumor immune responses and immunotherapy responses is not necessarily positive but it even could be negative. Therefore, the association between the tumor immune microenvironment (TIME) and immunotherapy response is complex, although the TIME has been recognized as a critical factor for responses to immunotherapy (Binnewies et al., 2018). Overall, tumors can be classified as “cold” (lack of immune infiltration) and “hot” (with abundant immune infiltration) types in terms of their TIME (Duan et al., 2020). It has formed a common sense that “hot” tumors respond better to immunotherapy than “cold” tumors (Galon and Bruni, 2019). However, our results do not appear to support this conclusion. Thus, further investigations into the association between the TIME and immunotherapy response are warranted.

To date, the conventional treatment strategies for cancer, including surgery, radiotherapy, chemotherapy, and targeted therapies, remain the major therapeutic approaches for most cancers. However, these strategies often achieve limited efficiency for advanced or recurrent cancers. Immunotherapy, particularly ICIs, has demonstrated success in treating various malignancies, including metastatic cancers, as a recently emerging treatment strategy. Nevertheless, the response rate of cancer patients to ICIs is around 20% to date (Cristescu et al., 2018). Thus, discovering predictive markers for immunotherapy response is significant. Several such markers used in current clinical practice include PD-L1 expression (Patel and Kurzrock, 2015), high tumor mutation burden (TMB) (Allgauer et al., 2018), and dMMR (Oliveira et al., 2019). However, these markers are not perfect in predicting immunotherapy response (Sun et al., 2020). For example, more than 50% of cancer patients with high expression of PD-L1 may not respond to ICIs (Reck et al., 2016). Therefore, identifying novel predictive markers for immunotherapy response is urgently needed. Our analysis suggests that the SFG mutation could be a positive predictor for immunotherapy response.

Among the proteins upregulated in SFG-mutated cancers, ACC (acetyl-CoA carboxylase) is a lipogenic enzyme to promote the synthesis of saturated fatty acids to meet the increasing demands of new membrane phospholipids in cancer cells (Igal, 2010). Thus, ACC is upregulated in various cancers, including breast, prostate, and liver cancers, and plays a critical role in cancer development (Wang C et al., 2010; Fang et al., 2014). It is justified that ACC is more abundant in SFG-mutated than in SFG-wildtype tumors since SFG mutations are associated with unfavorable clinical outcomes in various cancers. The lymphocyte-specific protein tyrosine kinase (Lck) is a member of the Src family of protein tyrosine kinases, whose upregulation may promote the development of diverse cancers, such as breast, colon, liver, and lung cancers (Sugihara et al., 2018; Bommhardt et al., 2019). Again, the positive association between SFG mutations and Lck expression conforms to the fact that the SFG mutation is an adverse prognostic factor in diverse cancers.

This study has several limitations. First, our results were obtained by the bioinformatics analysis. As a result, the associations between SFG mutations and various clinical and molecular characteristics in cancer are a correlation relationship but not a causal relationship. To demonstrate the causal relationship, further experimental and clinical studies are must. Second, in defining the phenotype of SFG mutations, we took the 119 key splicing factor genes as a whole but ignored the difference among the impact of different splicing factor genes’ mutations on splicing deregulation. Finally, in the single-cell data analysis, we defined the phenotype of SFG mutations based on the computational inference but not DNA sequencing, although such inference might result in inaccurate results.

In conclusion, the mutation in key splicing factor genes correlates with unfavorable clinical outcomes and disease progression, genomic instability, anti-tumor immunosuppression, and increased immunotherapy response in pan-cancer. Thus, the SFG mutation is an adverse prognostic factor and a positive marker for immunotherapy response in cancer.

## Data Availability

The original contributions presented in the study are included in the article/Supplementary Material, further inquiries can be directed to the corresponding authors.
